# Contrast with referrals to Tavistock and Portman Gender Identity Disorder Service

**DOI:** 10.1192/bjb.2018.110

**Published:** 2019-02

**Authors:** Katherine Rae Clyde

**Affiliations:** Consultant in Older Persons' Mental Health, Royal College of Psychiatrists; email: katie.clyde@nhs.net

This article shows a modest increase in referrals year on year, approximately 18%, with a majority of referrals received being for those assigned male at birth (AMAB). This is an interesting contrast with data from the Tavistock and Portman Gender Identity Disorder Service (GIDS) for children and adolescents.

Referrals to GIDS have increased from 97 in 2009/10 to 2016 in 2016/17. From 2014/15 to 2015/16, referrals increased by over 100% and from 2015/16 to 2016/17 they increased by 41%. Ages at referral seen by the service ranged from a very few at 3 to 17 years old.

Also in contrast to this much larger increase in referral rates is a marked change in the proportion of those assigned female at birth (AFAB). Up until 2011 there were more referrals of those AMAB. Since then the number of those AFAB referred has grown steadily, and in 2016/17 more than twice as many referrals were made for those AFAB as those AMAB (data available on GIDS website).

We need to be looking as a profession at these striking differences, and more research is required to determine the reasons for them. It may be that reducing stigma has led to higher referral rates, particularly among teenage girls but it could also be that the characteristics of those being referred are changing. This links with the finding that there seems to be a higher prevalence of autism spectrum conditions (ASC) in clinically referred, gender dysphoric adolescents than in the general adolescent population. Holt, Skagerberg and Dunsford (2014) found that 13.3% of referrals to the GIDS service in 2012 mentioned comorbid ASC (although this is likely to be an underestimate).

In this context, it is alarming that referral rates are increasing at a rate that services and research cannot keep up with. Both the American Academy of Pediatrics and the Australian Standards of Care and Treatment for Transgender and Gender Diverse Children and Adolescents appear to support both medical and surgical transition in adolescents. And yet long-term outcomes in this group are not known. We know that adults who have gender dysphoria and who transition report the dysphoria beginning in early childhood. We do not know yet know that those experiencing dysphoria in childhood will go onto experience dysphoria in adulthood. Indeed, we know that 80% of individuals referred to GIDS do not proceed to transition.

In the UK, we are fortunate to have a national service for children that follows the best available evidence, but there is an urgent need for both research and discussion. This is not always easy in a highly emotionally charged atmosphere.

